# Retraction: *In Silico* design of a multi-epitope vaccine for Human Parechovirus: Integrating immunoinformatics and computational techniques

**DOI:** 10.1371/journal.pone.0324093

**Published:** 2025-05-08

**Authors:** The PLOS One Editors

The *PLOS One* Editors retract this article [1] due to concerns about compromised peer review that call into question the validity of the reported results. We regret that the issues were not identified prior to the article’s publication.

All authors did not agree with the retraction.
